# Extracellular Hsp70 Reduces the Pro-Tumor Capacity of Monocytes/Macrophages Co-Cultivated with Cancer Cells

**DOI:** 10.3390/ijms21010059

**Published:** 2019-12-20

**Authors:** Elena Y. Komarova, Larisa V. Marchenko, Alexander V. Zhakhov, Alina D. Nikotina, Nikolay D. Aksenov, Roman V. Suezov, Alexander M. Ischenko, Boris A. Margulis, Irina V. Guzhova

**Affiliations:** 1 Laboratory of Cell Protection Mechanisms, Institute of Cytology of Russian Academy of Sciences, Tikhoretsky Ave. 4, St. Petersburg 194064, Russia; elpouta@yahoo.com (E.Y.K.); lelkavl@mail.ru (L.V.M.); nikotina.ad@gmail.com (A.D.N.); aksenovn@gmail.com (N.D.A.); roman.suezov@gmail.com (R.V.S.); margulis@incras.ru (B.A.M.); 2 Institute of Highly Pure Biopreparation of Federal Medical and Biological Agency of Russia, Pudozhskaya street, 7, St. Petersburg 197110, Russia; zhs2003@list.ru (A.V.Z.); ischenko@hpb-spb.com (A.M.I.)

**Keywords:** tumor microenvironment, eHsp70, carcinoma cells, THP1 monocytes, cytokines

## Abstract

Cancer cells are known to contain high levels of the heat shock protein 70 kDa (Hsp70), which mediates increased cell proliferation, escape from programmed cell death, enhanced invasion, and metastasis. A part of Hsp70 molecules may release from cancer cells and affect the behavior of adjacent stromal cells. To explore the effects of Hsp70 on the status of monocytes/macrophages in the tumor locale, we incubated human carcinoma cells of three distinct lines with normal and reduced content of Hsp70 with THP1 monocytes. Using two methods, we showed that the cells with knock-down of Hsp70 released a lower amount of protein in the extracellular medium. Three cycles of the co-cultivation of cancer and monocytic cells led to the secretion of several cytokines typical of the tumor microenvironment (TME) and to pro-cancer activation of the monocytes/macrophages as established by elevation of F4/80 and arginase-1 markers. Unexpectedly, the efficacy of epithelial–mesenchymal transition and resistance of carcinoma cells to anticancer drugs after incubation with monocytic cells were more pronounced in cells with lower Hsp70, e.g., releasing less Hsp70 into the extracellular milieu. These data suggest that Hsp70 released from tumor cells into the TME is able, together with the development of an anti-cancer immune response, to limit the conversion of a considerable part of monocytic cells to the pro-tumor phenotype.

## 1. Introduction

Tumor cells possess several attributes that are necessary to activate the immune response: They often carry mutant or improperly processed proteins that can trigger adaptive antitumor immunity, and inflammation often develops around the tumor area [1]. However, tumors can avoid an immune attack by converting the phenotype of the numerous cells that inhabit the tumor microenvironment (TME), including T-lymphocytes, macrophages, dendritic cells, and other members of the immune system [2]. Once in the tumor tissue, these cells begin to synthesize immunosuppressive cytokines, actions that lead to an increase in the concentration of myeloid-derived suppressor cells (MDSCs), activation of Fox3+ regulatory T-lymphocytes (Tregs), polarization of tumor-associated macrophages (TAMs) in the M2 phenotype, and suppression of T proliferation cells [3]. Tumor-associated macrophages, in addition to secreting tumor-promoting cytokines, support local inflammation, which, in turn, favors the genetic instability of tumor cells [4,5].

The list of cytokines whose concentrations increase in TME includes interleukin 1β (IL-1β), IL-6, IL-8, IL-10, transforming growth factor β (TGF-β), and tumor necrosis factor α (TNF-α) [6]. The pro-inflammatory cytokine, IL-1β, provokes the epithelial–mesenchymal transition (EMT) of tumor cells due to activation of the IL-1β/interleukin 1 receptor type 1 (IL-1RI)/β-catenin signaling pathway [7] and recruits additional immune cells to the tumor site [6]. Another proinflammatory cytokine, IL-6, is a resident of the TME in a large number of tumors [8,9,10,11]. IL-6, through its receptor IL-6R and Janus signaling pathway, activates STAT3, and this action leads to increased tumor cell proliferation, acquisition of resistance to anticancer drugs, EMT induction, and, ultimately, elevated metastasis [12,13]. Another factor that regulates the TME is TNF-α, a pleotropic cytokine that plays a key role in the regulation of apoptosis, tumor angiogenesis, inflammation, and immunity [14]. TNF-α is involved in almost all carcinogenesis stages and performs various functions depending on the tumor type and TME. At high concentrations, TNF-α correlates with tumor regression, and at long-term low concentrations, it contributes to tumor progression by stimulating the expression of other growth factors and cytokines [15]. One of the most studied TME factors, chemokine (C-C motif) ligand 2 (CCL2), also known as monocyte chemoattractant protein 1 (MCP-1), is expressed by tumor cells, and its expression correlates with poor prognosis in most human tumors [16,17]. CCL2 also recruits MDSCs to tumors [18].

An important player in TME is heat shock protein 70 kDa (Hsp70). Myriad tumors of different histogenesis often exhibit elevated Hsp70 expression [19]. Hsp70 is a multifunctional protein that can protect tissues from proteotoxic shock and the whole organism from various types of pathogens (microbes/viruses/oncogenes). Hsp70 can recognize abnormally folded or mutant protein and decide its fate: Proteolysis or refolding [20]. Hsp70 is released from tumor cells [21] and can activate innate and adoptive antitumor [22,23,24]. However, it is unclear whether and how extracellular Hsp70 can affect the cells in TME, particularly TAMs.

Any solid tumor has a specific volume, and the naive macrophage that migrates inside the tumor body will meet progressively newer tumor cell populations. The aim of this study was to understand how the macrophage phenotype changes as it moves through new populations of tumor cells with high or low extracellular Hsp70 (eHsp70), how the cytokine profile of the TME varies, and how tumor cells react to these actions.

## 2. Results

### 2.1. Hsp70 Knock-Down in Carcinoma Cells Reduced the Amount of Released Chaperone

**Figure 1:**
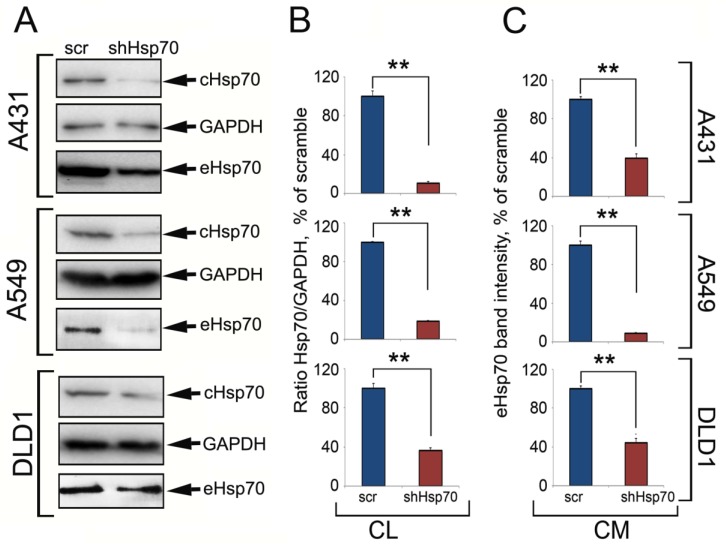
Tumor cells with high Hsp70 expression released more Hsp70 into the extracellular space than tumor cells with down-regulated Hsp70. (**A**) Western blot of cell lysates (CL) from three carcinoma cell lines after Hsp70 knockdown and eHsp70 trapping with ATP-Agarose from conditioned medium (CM). (**B**,**C**) The intensity of bands from (**A**) was measured with TotalLab software. ** *p* < 0.01.

To understand how the Hsp70 expression level in tumor cells influences the chaperone content in the TME (represented in our study by conditioned culture medium), we generated human carcinoma cells with downregulated Hsp70 expression using specific small hairpin RNA (shRNA). The control cells were infected with a lentivirus with the gfp gene (scr). These cells were designated as A431scr and A431shHsp70, A549scr and A549shHsp70, and DLD1scr and DLD1shHsp70. Hsp70 expression levels in three cell pairs were evaluated with western blotting. The amount of eHsp70 in conditioned medium was measured using micro-chromatography on ATP-agarose coupled with western blotting as well as an ATP-enzyme linked immunosorbent assay (ELISA). shHsp70 lentivirus infection reduced the Hsp70 level to 10.53% ± 1.59% for A431 cells, 18.52% ± 3.54% in A549 cells, and 36.23% ± 1.66% for DLD1 cells (all compared to control levels; Figure 1A,B). Using an ATP-ELISA, eHsp70 was two-fold higher in A431scr compared to A431shHsp70 (33.6 ± 2 ng/mL versus 17.2 ± 2.0 ng/mL), 2.9-fold higher in A549scr compared to A549hsHsp70 (30.0 ± 1.5 ng/mL versus 10.4 ± 1.2 ng/mL), and two-fold higher for DLD1scr compared to DLD1shHsp70 (41.0 ± 1.2 ng/mL versus 20.4 ± 2.2 ng/mL). The ATP-chromatography and western blot data correlated with the ATP-ELISA results (Figure 1). Thus, the higher the level of intracellular Hsp70, the higher the eHsp70 in the conditioned medium.

### 2.2. The TME Cytokine Profile Depended on Tumor Cells

In order to model the changes in the TME cytokine profile as the macrophage/monocyte progressed inside the tumor, we created an in vitro system where we allowed physical contact between the tumor and monocytic cells. We employed THP1 cells that are widely used in studies on macrophage-M2 transition mechanisms [25,26] and tested their ability to change the phenotype under the action of certain cytokines. The data of immunoblotting showed that being treated with phorbol myristate combined with IFN-γ meant cells approached the M1 phenotype and the level of F4/80 was significantly reduced. On the contrary, after treatment with “CellXVivo Human M2 Macrophage Differentiation Kit”, the level of F4/80 in THP1 cells increased significantly, suggesting that their phenotype can be regulated by the tumor secretome (Figure S1).

Next, we performed a three-stage co-cultivation of tumor cells (with normal or reduced Hsp70) with THP1 cells; each time, the “educated” THP1 cells were transferred to fresh tumor cells culture (see Figure S2).

**Figure 2:**
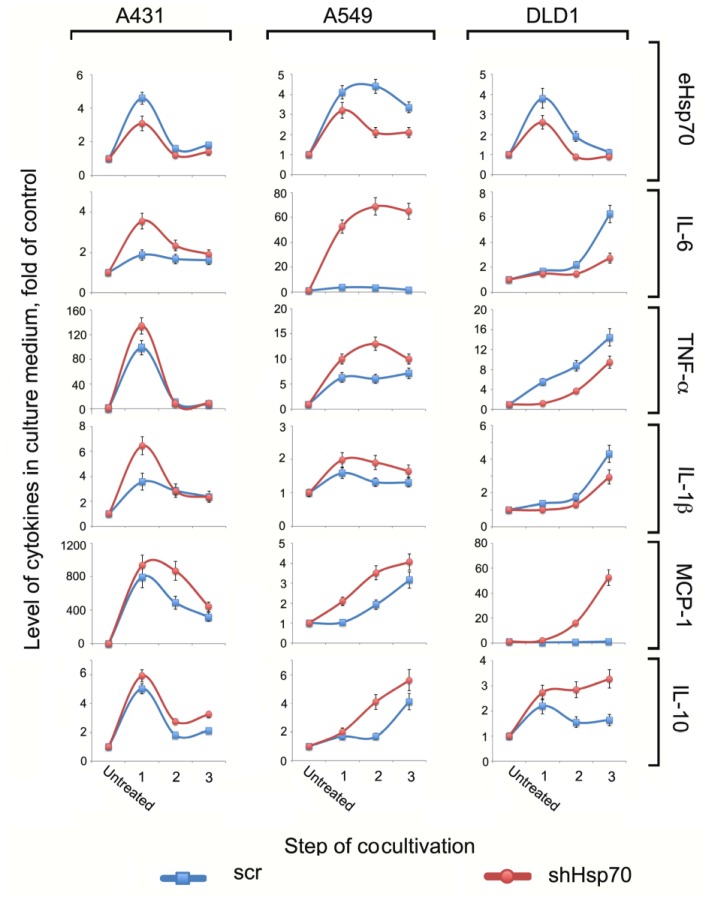
Exogenous Hsp70 and cytokine profiles after co-cultivation of carcinoma cells with normal and downregulated Hsp70 c monocytic THP1 cells. Conditional medium from carcinoma cells collected after co-cultivation with THP1 cells (stages 1, 2, 3) analyzed with magnetic-bead-based multiplex immunoassay and MilliPlex technology. Levels of eHsp70 in culture medium were measured with the aid of the ATP-ELISA method.

First, we measured eHsp70, IL-1β, TNF-α, IL-6, MCP-1, and IL-10 levels in the conditioned media after each co-cultivation step. Overall, the cytokine profile was unique for each cell line, although there were certain observable patterns (Figure 2). For example, in A431 and A549 cells, all cytokine levels were higher when the cells expressed reduced Hsp70 (and thus produced less chaperone in extracellular milieu). However, in DLD1 cells, pro-inflammatory cytokine levels were higher in cells with normal compared to reduced Hsp70 levels. Interestingly, the level of MCP-1, the cytokine responsible for recruiting fresh macrophages to the tumor lesion, and pro-tumor IL-10 were higher when DLD1shHsp70 cells were used rather than the cells with a normal Hsp70 level. The level of eHsp70 opposite was higher in the culture medium of A431scr, A549scr, and DLD1scr cells, which underwent three stages of co-cultivation (Figure 2, upper panel).

### 2.3. Tumor Cell-Induced Macrophage “Education”

**Figure 3:**
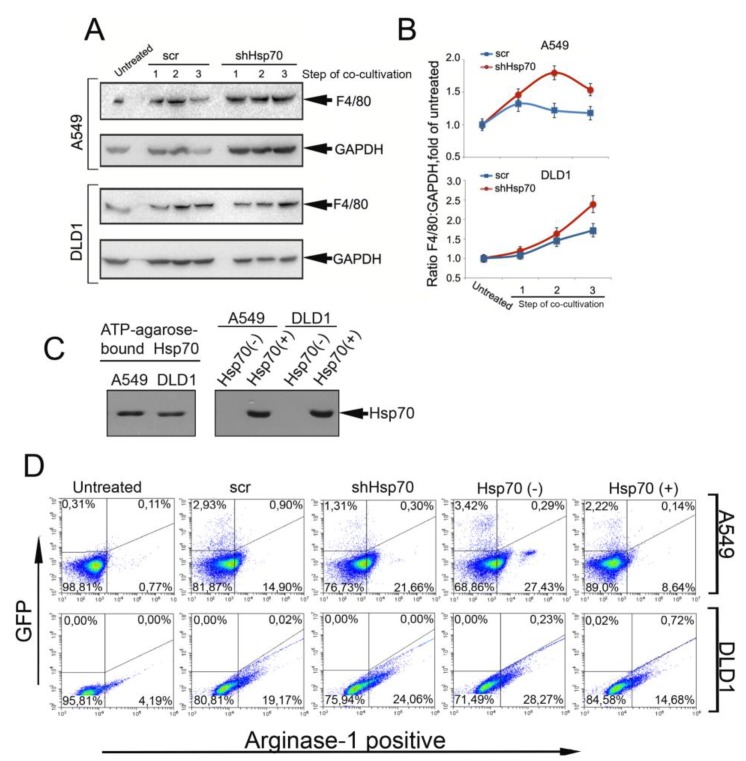
THP1 monocytes acquired pro-tumor properties when co-cultivated with tumor cells. (**A**) Western blotting of THP1 cells after co-cultivation with carcinoma cells with normal and downregulated Hsp70. (**B**) Intensity of protein bands from A was measured with TotalLab software. (**C**) Western blotting analysis of Hsp70 attached to ATP-agarose during co-cultivation of A549scr or DLD1scr cells with THP1 cells (“Hsp70(−)”) (left panel). Conditioned media from Hsp70(−) and Hsp70(+) probes were analyzed with the aid of western blotting (right panel). (**D**) THP1 cells were incubated with A549 and DLD1 cells, “scr” and “shHsp70”, and stained with antibody against arginase-1. “Hsp70(−)” samples were prepared with the aid of ATP-agarose that was added to co-cultivation medium for the whole time of co-cultivation. “Hsp70(+)” samples were prepared as follows: human rHsp70 was added to A549scr or DLD1scr cells 18 h before the THP1 cells to induce the release of intracellular chaperone and then THP1 cells were introduced to co-culture.

To determine whether cytokine and eHsp70 profile modulation in the TME is associated with the pro-tumor conversion of monocytic THP1 cells, we examined the expression of the F4/80 and arginase-1 markers using western blotting and flow cytometry. THP1 cell probes were taken after each stage of co-cultivation with A549 and DLD1 carcinoma cells. Irrespective of the intracellular or extracellular Hsp70 content in the carcinoma cells, the F4/80 and arginase-1 level increased during co-cultivation. However, in both carcinoma cells, the pro-tumor markers’ level was higher in co-culture with cells with the reduced Hsp70 (Figure 3A,B, Figure S3).

To prove that the transition of monocytic cells to a pro-tumor phenotype is closely associated with the level of eHsp70 in the TME, in addition to the experiments described above, we varied the level of eHsp70 in the co-cultivation medium. To reduce the eHsp70 level, we performed the co-cultivation of A549scr and DLD1scr cells in the presence of ATP-agarose, which is used in the purification of the protein. Alternatively, to increase the Hsp70 amount in the culture medium, we employed the technique described elsewhere [21]. Briefly, pure recombinant Hsp70 was added to the A549scr and DLD1scr cells for 18 h before the introduction of THP1 cells; the addition of exogenous Hsp70 caused the release of its endogenous analogue [21], the amount of which was also estimated with the aid of chromatography on ATP-agarose followed by western blotting.

According to the results of the immunoblotting, the eHsp70 was successfully captured with ATP-agarose in the “Hsp70(−)” probe. The addition of the pure chaperone to tumor cells led to the powerful release of endogenous analogue, which is consistent with our early results (Figure 3C) [21].

The analysis of arginase-1 in THP1 cells incubated with cancer cells showed that it was higher after co-cultivation with A549shHsp70 and DLD1shHsp70 cells compared with “scr” cells (21.66% vs. 14.9% for THP1 cells incubated with A549sHsp70 and A549scr, respectively, and 24.06% vs. 19.17% for the DLD1 couple, see Figure 3D). The removal of Hsp70 from the media with the aid of ATP-agarose led to an elevation of the quantity of arginase-1-positive cells in the THP1 cell population up to 27.43% vs. 14.9% and 28.27% vs. 19.17% for A549 and DLD1 cells, respectively. The elevation of eHsp70 in the co-incubation medium led to a reduction of arginase-1-positive THP1 cells in both A549 and DLD1 cells (Figure 3D).

Interestingly, primary human monocytes incubated with carcinoma cells produced different amounts of eHsp70 in the medium, and demonstrated an even more impressive difference between “scr” and “shHsp70” cells compared with naïve THP1 cells (Figure S4). Importantly, the level of intracellular Hsp70 was similar in “Contr”, “Hsp70(−)”, and “Hsp70(+)” cancer cells, suggesting that monocyte phenotype modulation was linked mainly to exogenously persisting chaperone (Figure S5).

Collectively, these data suggest that higher eHsp70 can protect monocytic cells from conversion to a pro-tumor phenotype.

### 2.4. Co-Cultivation with Monocytic THP1 Cells Stimulated Carcinoma Cell Aggressiveness

**Figure 4:**
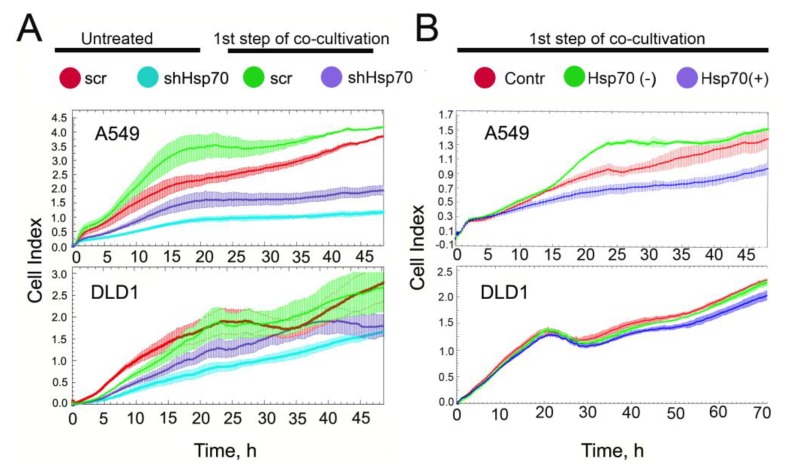
Proliferation of tumor cells increased after co-cultivation with THP1 cells. (**A**) Untreated (red and blue curves) A549scr and A549shHsp70 and DLD1scr and DLD1shHsp70 cells and that after one co-cultivation step with THP1 monocytes (green and violet), were seeded on E-plates. Recording with xCELLigence equipment commenced when cancer cells attached to the bottom of the plates and lasted for 48 or 72 h. (**B**) The proliferation capacity of A549scr and DLD1scr cells treated as described above for “Hsp70(−)” and “Hsp70(+)” samples after co-cultivation with THP1 monocytes.

THP1 monocytes that contacted cancer cells with variable Hsp70 content and a distinct ability to export eHsp70 changed their phenotype to pro-tumorigenic. These data suggest that the resulting macrophages can also affect the behavior of their tumor partners. In order to prove such an acquired property, we compared growth dynamic parameters for A549scr and A549shHsp70 and DLD1scr and DLD1shHsp70 cells after co-cultivation with THP1 cells using xCELLigence technology. As expected, the cells with reduced Hsp70 (blue curve) grew more slowly; the growth index decelerated approximately 3.5-fold in A549shHsp70 compared to A549scr cells (red curve) and 1.7-fold in DLD1shHsp70 compared to DLD1scr cells (Figure 4A).

Co-cultivation with THP1 cells increased proliferation in all cell groups regardless of the Hsp70 expression level: A549scr cells grew 1.6-fold faster (green curve), whereas A549shHsp70 cells grew 2-fold faster after incubation with THP1 cells (violet curve). There was a similar acceleration in cell growth after co-cultivation observed in DLD1scr and DLD1shHsp70 cells (1.5-fold and 1.8-fold, respectively; Figure 4A, low panel).

We also estimated the proliferation capacity of carcinoma cells after a manual alteration of the eHsp70 amount in the co-cultivation milieu as described above. The depletion of Hsp70 from culture medium (“Hsp70(−)”, green curve, Figure 4B) made A549scr cells grow faster than “Contr” A549scr cells whereas the presence of a high amount of eHsp70 in the culture medium (“Hsp70(+)”, violet curve, Figure 4B) considerably slowed cell growth. Changes in growth rates of DLD1 cells associated with the regulation of eHsp70 content were less evident (Figure 4B, lower panel).

We can conclude that co-incubation with monocytic cells accelerated the growth of tumor cells; when the eHsp70 content was lower, the cells grew slightly faster. A similar trend was well-maintained at subsequent co-cultivation stages (data not shown).

**Figure 5:**
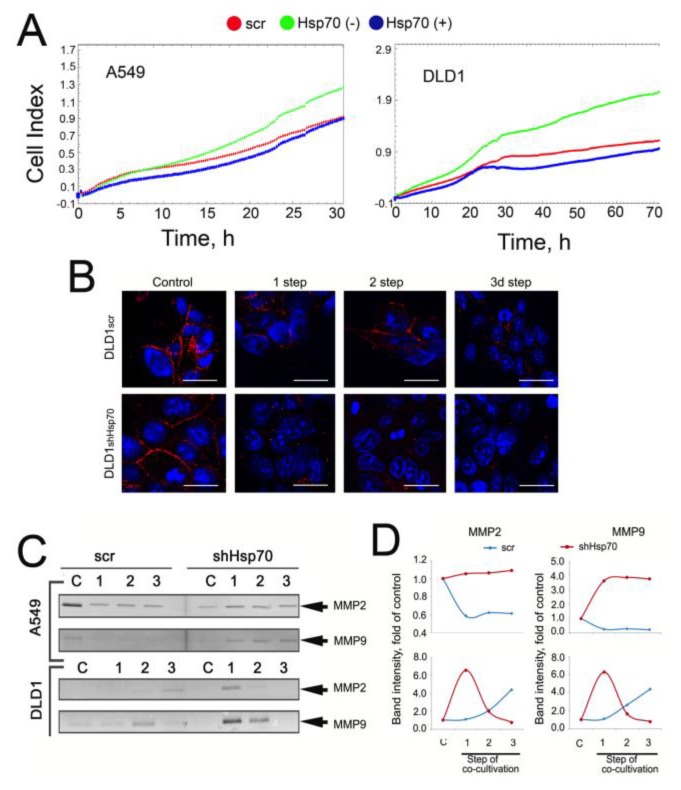
Reduced quantity of eHsp70 in culture media of cancer cells leads to a greater EMT after co-cultivation with monocytic cells. (**A**) Migration assay. A549scr and DLD1scr cells were treated as described above and then were seeded to CIM plates of xCELLigence equipment. Recording lasted 32 h for A549 cells and 72 h for DLD1 cells. (**B**) DLD1scr and DLD1shHsp70 cells were seeded on cover glasses and subjected to three steps of co-cultivation with THP1 cells. After each step, cells were stained with an antibody against E-cadherin (red). Nuclei were stained with DAPI (blue). Scale bars are 10 μM. (**C**) MMP2 and MMP9 levels were evaluated with zymography. (**D**) The intensity of MMP2 and MMP9 bands was measured with TotalLab software.

Another important indicator of tumor aggressiveness, migration capacity, was estimated using a migration assay with the aid of xCELLigence technology. A549scr or DLD1scr cells were co-cultivated with THP1 cells for 24 h in media with an elevated quantity of eHsp70 (“Hsp70(+)”) or alternatively, with a reduced amount of chaperone (“Hsp70(−)”), as described in the previous section. After one co-cultivation circle, THP1 cells were removed, and cancer cells were collected and seeded on CIM plates of an xCELLigence apparatus. The removal of eHsp70 from co-cultivation medium made cancer cells migrate faster (approximately 45% faster for A549 cells and two-fold faster for DLD1 cells). The elevation of eHsp70 in the culture medium decelerated the migration rate in both cell lines (Figure 5A).

We noticed that co-cultivation with THP1 monocytes caused the loss of intercellular contacts in DLD1 cells, probably due to the reduction of E-cadherin localized to the plasma membrane. Confocal microscopy confirmed our supposition; it showed that the E-cadherin disappearance was more profound in DLD1shHsp70 cells (Figure 5B).

The ability to form metastases relates to the activity of matrix metalloproteinases (MMPs), most often MMP2 and MMP9. We tested their activity in A549scr, A549shHsp70, DLD1scr, and DLD1shHsp70 cells subjected to co-cultivation with THP1 cells. Using zymography, we analyzed the MMP2 and MMP9 content in serum-free medium collected from tumor cells 12 h after the removal of THP1 cells at each co-cultivation step. The levels of both MMPs were higher in cells with downregulated Hsp70. Interestingly, in DLD1 cells, the highest MMP levels were observed at the first co-cultivation step and subsequently declined. In DLD1scr cells, however, the levels were slightly elevated with each co-cultivation step (Figure 5C,D). In A549scr cells, the level of both MMPs decreased for each co-cultivation step while in A549shHsp70 cells, it was elevated compared to untreated cells (Figure 5C,D). Overall, this data demonstrated that low Hsp70 levels, which leads to low eHsp70, correspond to a more aggressive phenotype for carcinoma cells.

### 2.5. A549 Cells Acquired Drug Resistance after Incubation with Monocytes

**Figure 6:**
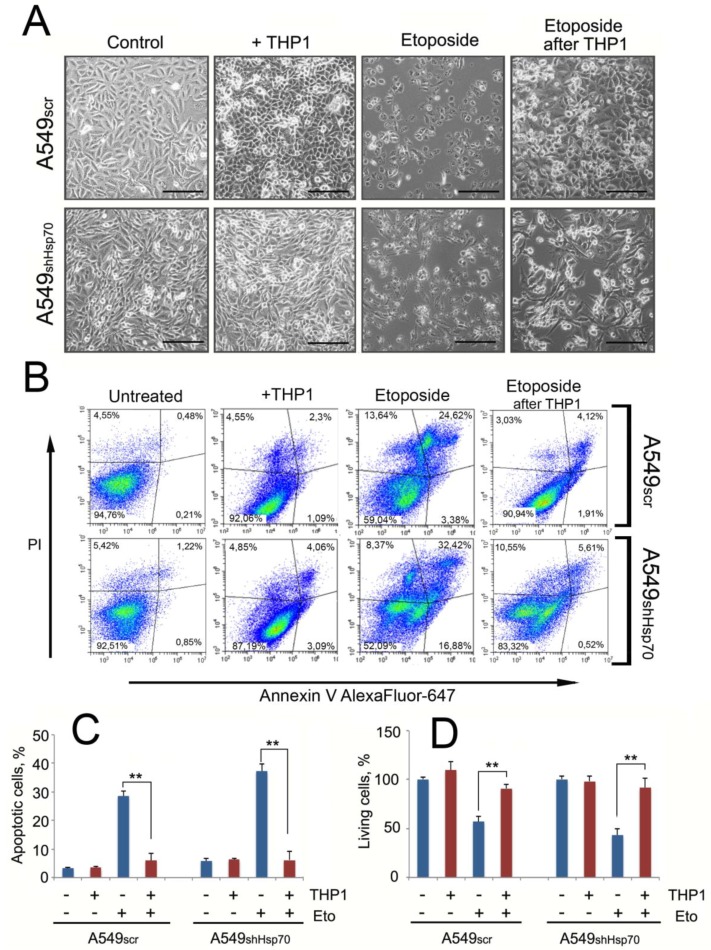
Co-cultivation of A549scr and A549shHsp70 cells with ThP1 cells led to tumor cell resistance to the anti-cancer drug etoposide. (**A**) A549scr and A549shHsp70 were co-cultivated with THP1 cells for 24 h. After THP1 cells were removed, 20 μM of etoposide was added. Micrographs of the carcinoma cells are shown. Scale bars 100 μm. (**B**,**C**) A549 cells with normal and downregulated Hsp70 were co-cultivated with THP1 cells and then were treated with etoposide (Eto). (**B**) Density plot of one representative experiment; (**C**) Percentage of apoptotic cells was measured with flow cytometry. Data from three independent experiments are summarized. ** *p* < 0.01. (**D**) Total amount of A549 cells co-cultivated with THP1 cells and survived after treatment with Eto was evaluated with cell counting using LUNA2 equipment. ** *p* < 0.01.

To determine whether monocytes may affect the sensitivity of tumor cells to anticancer drugs, we incubated A549scr and A549shHsp70 cells with 20 μM etoposide for 24 h following a cycle of co-cultivation with THP1 cells (Figure 6A). Treatment with etoposide caused massive apoptosis in A549scr cells (28.6% ± 1.6% of total cells), and in A549shHsp70 this value was significantly higher (47.3% ± 2.6%). After co-cultivation with monocytic cells, the number of apoptotic cells was reduced to 5.98% ± 2.43% and 6.19% ± 3.1%, respectively, in A549scr and A549shHsp70, data that suggest monocyte-mediated stimulation of lung tumor cell protection (Figure 6B). The apoptosis data were confirmed by measuring cell viability. Etoposide administration reduced the cell population to 61.2% ± 5.2% for A549scr cells and to 43.25% ± 6.4% for A549shHsp70 compared to control cells (Figure 6C). However, in cells induced with etoposide after contact with THP1 cells, the total number of cells was similar to the control (Figure 6C).

### 2.6. Delivery of Recombinant Hsp70 within Melanoma Tumor Hampers Maturation of Pro-Cancer Macrophages

To support the data obtained in vitro, we explored how the elevation of Hsp70 content in the TME affects the transition of macrophages to the M2 phenotype and treated B16 melanoma-bearing mice with Hsp70-containing hydrogel. Recently, we demonstrated that Hsp70 applied on the tumor surface was able to penetrate melanoma cells and pull out its cellular analogue into the TME, causing significant delay in tumor progression [21,27]. In the present study, C57Bl/6 mice were subcutaneously injected with 1 × 10^6^ B16 cells and the application of Hsp70-containing gel or control gel (vehicle) was performed onto preliminarily shaven areas of skin surrounding a 7-day-old B16 tumor; the application was repeated every 3 days until the end of experiments. Tumors were derived on day 19 and used for histological study. The slices were stained with antibodies against arginase-1.

**Figure 7:**
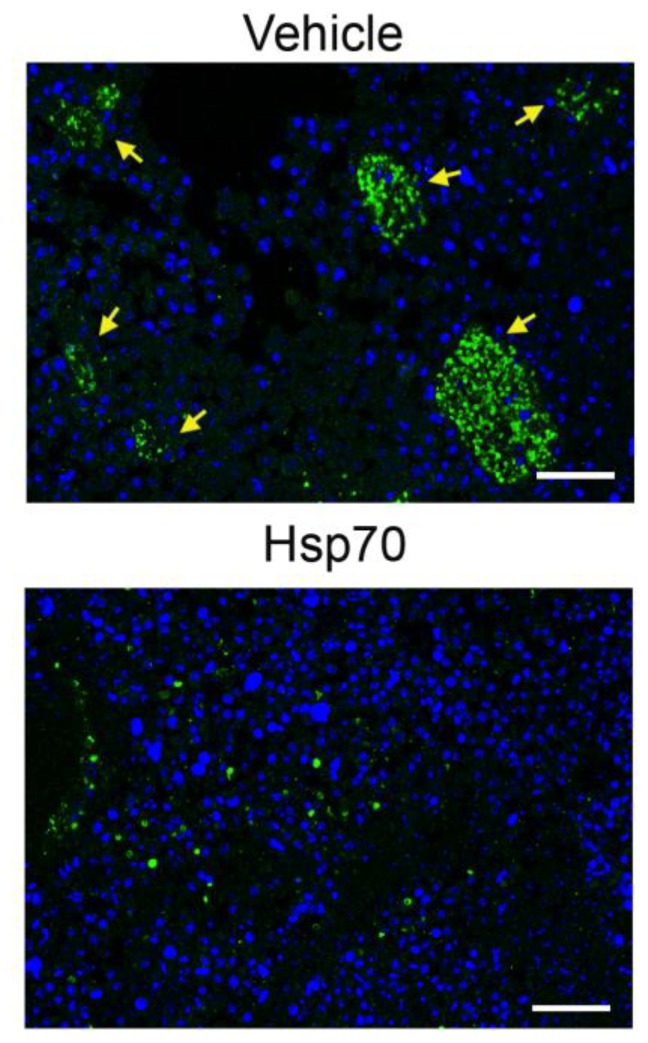
Hsp70-based composition applied to a growing B16 melanoma tumor in vivo leads to a reduction of arginase-1-positive macrophages in the tumor locale. The animals were subcutaneously injected with 10^6^ B16 cells. The application of Hsp70-containing gel or control gel (vehicle) was performed onto preliminarily shaven areas of skin surrounding a 7-day-old B16 tumor; the application was repeated every 3 days until the end of experiments. Tumors were derived on day 19 and histological slices were stained with antibodies against arginase-1 (green), nuclei were stained with DAPI (blue). Scale bars: 100 µm. Yellow arrows indicate M2 macrophage colonies.

Tumors treated with control gel were infiltrated with large colonies of arginase-1-positive macrophages, each colony contained approximately 50 to 200 cells, whereas the treatment with Hsp70-containing gel led to a significant decrease in arginase-1-positive macrophages in the tumor locus, and they were represented by single cells that did not form colonies, suggesting that the reduction of eHsp70 in the TME leads to enhanced recruitment of M2-type macrophages (Figure 7).

## 3. Discussion

Tumor cells often possess high levels of chaperones, particularly Hsp70, that upregulate their proliferation and mediate anti-growth signal evasion, escape from programmed cell death, avoidance of cell senescence, cell invasion, and metastasis [28]. On the contrary, Hsp70-based protection can be reversed by triggering its export from cancer cells. Such export to the outer surface of the plasma membrane and extracellular matrix can be initiated by subjecting tumor cells to physical factors like irradiation or to drug-like chemicals [29,30,31]. Recombinant eHsp70 administrated intratumorally activates both innate and specific anticancer immune response in tumor-bearing animals [23,24] due to the capacity of the chaperone to pull out its endogenous analogue from the tumor [21]. Since tumor cells can release Hsp70 into the extracellular milieu without an additional trigger [32], one could suggest the higher the level of endogenous Hsp70 expression in tumor cells, the more chaperone molecules would appear in the TME. We selected three carcinoma cell lines with elevated Hsp70 levels and used two high-resolution assays to show that shRNA-mediated Hsp70 downregulation reduced eHsp70; the level of intracellular chaperone correlated well with that released into the extracellular milieu (Figure 1).

Hsp70 release into the TME can provoke a number of effects on both cancer and stromal cells by inducing the secretion of certain cytokines [33,34]. By analyzing the cytokine profile from the conditioned media produced by co-cultivating cancer cells with different Hsp70 levels and monocytic cells, we observed marked variability in the number and rate of pro-inflammatory cytokines independent of whether tumor cells with normal or reduced Hsp70 contacted the THP1 cells. However, the levels of pro-tumor cytokines, namely IL-10 and MCP-1, were obviously higher when cells with downregulated Hsp70 contacted monocytes for all three carcinoma cell pairs. In other words, the lower the eHsp70 in the conditioned medium, the higher the secreted pro-tumor cytokine concentrations (Figure 2). eHsp70 has chaperone- and cytokine-regulating activity (chaperokine) and can stimulate anti-tumor immune responses [35]. The results of the present study demonstrate that in the combination of macrophage-like and tumor cells, eHsp70 may play an anti-tumor role.

Macrophages constitute the TME cellular population whose phenotype is affected by adjacent tumor cells; they can strongly influence cancer cell tumorigenicity [36]. When they are stimulated by certain cytokines and acquire a TAM with F4/80 expression [37], macrophages contribute to tumor cell proliferation by secreting specific growth factors [38]. In our experiments, the transition of monocytic cells to the pro-tumor phenotype, denoted by increased F4/80 and arginase-1 expression, occurred more efficiently when the monocytes were incubated with A549 and DLD1 cells with downregulated Hsp70 (i.e., when the eHsp70 content in the extracellular space was lower; Figure 3). The experiments with modulation of the amount of eHsp70 in the co-cultivation medium confirmed our hypothesis that the quantity of eHsp70 is responsible for the phenotype of monocytic cells. The relationship between Hsp70 export and macrophage differentiation and maturation was previously reported by several groups [39,40]. In all cases, the eHsp70 increase was concomitant with the larger effect on the macrophage phenotype.

The capacity of carcinomas to recruit and activate TAMs largely depends on the malignant cells attaining an undifferentiated mesenchymal phenotype as part of the so-called EMT [41,42]. Tumor cell proliferation increased after their contact with monocytic cells and was higher in Hsp70-knockdown tumor cells as well as in the absence of eHsp70 in co-cultivation medium (Figure 4). Co-cultivation of tumor cells with THP1 enhanced the migration of both A549scr and DLD1scr when eHsp70 was removed from the cell medium (Figure 5A). In cells with reduced Hsp70, intercellular contacts were disturbed. Examination of E-cadherin expression revealed that it disappeared to a greater extent when tumor cells with downregulated Hsp70 were co-cultivated with monocytes, data that show Hsp70 knockdown leads to a more rapid and effective EMT (Figure 5B). Our data correlate well with a very recent report by Kasioumi et al. [43], who demonstrated that Hsp70 regulates the steps of metastasis, including EMT and migration. The absence of Hsp70 in cancer cells appears to be the destruction of the Hsp70-dependent heterocomplexes of E-cadherin/catenins, which function like an anchor between neighboring cells [43]. Liu et al. demonstrated that Hsp70 inhibits high-glucose-induced EMT by modulating Smad expression and activation in rat peritoneal cells [44].

Another feature of metastasis is excessive extracellular matrix degradation, mediated predominantly by MMPs [45,46,47]. In our experiments, MMP levels correlated with the F4/80 and arginase-1 expression data (Figure 3). Further, MMP levels were higher in A549shHsp70 and DLD1shHsp70 cells compared with A549scr and DLD1scr cells, respectively (Figure 5D,E).

Finally, we noticed that co-cultivation of tumor cells with monocyte-like cells led to the resistance of tumor cells to etoposide. A significant number of A549scr cells underwent apoptosis, and as expected, A549shHsp70 demonstrated significantly greater sensitivity to the antitumor drug. After co-cultivation with THP1 cells, both A549 cells with normal and reduced Hsp70 levels demonstrated similar etoposide resistance (Figure 6).

To approve the data obtained in vitro, we examined the amount of TAMs inhabiting B16 melanoma tumors that were treated with exogenous Hsp70; in this experiment, we showed that chaperone delivery caused significant reduction of pro-cancer macrophages and resorption of multicellular colonies formed by arginase-1-positive cells in the absence of Hsp70 (Figure 7). Interestingly, Lopes et al. reported that the bacterial homologue of Hsp70, DnaK, was able to promote IL-10-dependent transition of mouse macrophages to a pro-tumor phenotype. However, this conversion did not occur in the context of the existing TME [48]. Thus, Hsp70 releasing from cells of TME can play different roles in pro-cancer maturation of macrophages inhabiting the tumor niche.

The cytoprotective role of Hsp70 in cancer cells is well established [49,50,51], and the results presented here provide new insight into Hsp70’s function in the extracellular space. Our data indicate that cells with reduced Hsp70 content after prolonged physical contact with monocytes/macrophages became more aggressive than their “normal” counterpart. We suggest that the role of Hsp70 release into the TME may help regulate macrophages that belong to two different pools, namely pro-inflammatory and pro-tumor. The first possibility relates to eHsp70 activity as a damage-associated molecular pattern (DAMP), which was recently affirmed by two groups [52,53]. The second possibility is still questionable, and here, we report some additional data that favor the hypothesis that Hsp70 could participate in macrophage sorting in the tumor.

## 4. Materials and Methods

### 4.1. Cells

Human lung carcinoma A549 cells, human leukemic THP1 monocytes, and human carcinoma A431 cells were obtained from the Russian Collection of Cell Cultures (Institute of Cytology, Russian Academy of Sciences, St. Petersburg, Russia). Human colon carcinoma DLD1 and HCT-116 cells were kindly provided by Dr. N. Barlev (Institute of Cytology of Russian Academy of Sciences, St. Petersburg). All cells were grown in Dulbecco’s modified eagle’s medium (DMEM; Lonza, Basel, Switzerland), with 10% fetal calf serum (Paneco, Russia), penicillin G (100 ME/mL), and streptomycin (100 μg/mL) (Biolot, Russia). Cells were incubated in a humidified incubator at 37 °C with 6% CO_2_.

Vector plasmids for Hsp70 knockdown were purchased from EuroGene (Moscow, Russia). Sequences for shRNA to HSPA1A (Hsp70) were mature antisense, TTGATGCTCTTGTTCAGGTCG; scrambled RNA, TAATACGACTCACTATAGGG. Packing (D8.91) and envelope (pVSV-G) plasmids were kindly provided by Dr. L. Glushankova (Institute of Cytology of Russian Academy of Sciences, St. Petersburg). The HEK-293T cells were transfected using polyethylenimine (PEI) with a mixture of all three plasmids. Cells were selected with puromycin (2.0 μg/mL; Sigma, St.Loise, MO, USA) at least 2 weeks prior to the start of the experiment.

### 4.2. Western Blot

Hsp70 knockdown in A549 and DLD1 cells was verified with western blotting. The cells were lysed on ice in 20 mM Tris-HCl (pH 7.5), 20 mM NaCl, 0.01% Triton X-100, 1 mM EDTA, and 1 mM PMSF. Equal amounts of total protein (20 μg/lane) were electrophoresed with a 10% sodium dodecyl sulfate (SDS) polyacrylamide gel. Protein was transferred to a polyvinylidene difluoride (PVDF) membrane, and non-specific binding on the membrane was blocked with 5% fat-free milk in phosphate-buffered saline (PBS). Membranes were further incubated with monoclonal anti-Hsp70 antibody (Clone 3C5, [54]). Glyceraldehyde 3-phosphate dehydrogenase (GAPDH) was employed as a loading control (Abcam, UK). To examine the expression of TAM markers in THP1 cells after incubation with tumor cells, western blotting utilized an antibody against F4/80 (Abcam, Cambridge, UK).

### 4.3. Analysis of Hsp70 Release from Living Cells

A549scr, A549shHsp70, DLD1scr, and DLD1shHsp70 cells were seeded in 75-cm^2^ flasks. When the monolayer reached 75% confluence, cells were washed with PBS and placed into serum-free medium for 2 h. The conditioned medium was supplemented with Tween-20, Tris-HCl (pH 7.5), and MgCl_2_ to reach final concentrations of 0.1%, 20, and 5 mM, respectively. Medium was mixed with ATP-Agarose gel (Sigma-Aldrich, St.Loise, MO, USA), and the suspension was rotated overnight at 4 °C. The slurry was washed with the application buffer, mixed with an equal volume of 2x buffer for electrophoretic samples that contained 4% SDS, and subjected to electrophoresis and immunoblotting using the anti-Hsp70 monoclonal antibody (Clone 3C5).

### 4.4. ATP-ELISA

The ATP-ELISA was performed as described elsewhere [55] with some modifications. Briefly, an ATP-albumin complex was immobilized on the wells of 96-well EIA/RIA Costar 3590 (Corning, USA) plates. Culture media after co-cultivation of A431, A549, and DLD1 cells with normal and downregulated Hsp70 with THP1 cells were spun at 10,000× *g* for 10 min and Tris-HCl, pH7.5, NaCl, MgCl_2_, and Tween-20 were added to reach final concentrations 20 mM, 140 mM, 5 mM, and 0.05%, respectively. Buffer containing 20 mM Tris-HCl, pH7.5, 140 mM NaCl, 5 mM MgCl_2_, and 0.2% Tween 20 was then used for blocking as well as for all subsequent steps and washes. Culture media and calibration standards of pure human recombinant Hsp70 were applied to the wells overnight at 4 °C. After that, wells were washed, and antibody raised in a rabbit immunized with the recombinant human Hsp70-ATP complex was added followed by goat anti-rabbit antibody conjugated with horseradish peroxidase (Jackson ImmunoResearch Laboratories, Cambridge, UK) and hydrogen peroxide was used to develop the calorimetric reaction. Optical density was measured at 450 nm using Fluorofot ‘Charity” microplate reader (Probanauchpribor, Russia. Hsp70 concentration in samples was determined using the calibration curve (5–250 ng/mL) obtained from the titrated standards.

### 4.5. Experiments on Macrophage “Training”

In order to mimic the process of the macrophage moving inside the tumor tissue, we applied an in vitro model of phased macrophage “training”. A549scr, A549shHsp70, DLD1scr, and DLD1shHsp70 cells were seeded in 10-cm Petri dishes at 6.0 × 10^4^ cells/mL the evening before the experiment. After the cells attached to the bottom of the dish, THP-1 cells were applied in a 1:1 ratio. Co-cultivation was performed for 24 h, after which the trained macrophages (stage 1) were transferred to fresh tumor cells that were seeded the day before in similar proportions (Figure S2). In order to maintain the 1:1 ratio at all stages of co-cultivation, at the first stage, co-cultivation was performed in three Petri dishes, two dishes for the second, and one dish for the third. Suspended THP1 cells were harvested by pipetting. At each subculture, the number of dead cells in the culture was counted using trypan blue staining. After each stage, carcinoma cells, THP1 cells, and the conditioned media in which they were co-cultivated were harvested separately, frozen, and stored at −80 °C.

### 4.6. Cytokine Measurement

Interleukin 1β (IL-1β), IL-6, MCP-1, TNF-α, and IL-10 levels in the conditioned medium after each of the three cell co-cultivation stages were evaluated using multiplex immunological analysis on magnetic microspheres and MilliPlex technology according to the manufacturer’s recommendations (Merck, Germany). The Milliplex Human Cytokine Magnetic Panel HCYTOMAG-60K was kindly provided by Merck. Two independent measurements of the cytokine profile of the conditioned medium were performed.

### 4.7. Purification of Human Recombinant Hsp70

Human recombinant Hsp70 was purified from *Escherichia coli* cells transformed with pMSHsp70 plasmid and detoxified with the use of polymyxin B–agarose gel (Sigma, St.Loise, MO, USA) as described elsewhere [56] The concentration of bacterial lipopolysaccharide in the final preparation was lower than 0.1 MU/mL, according to LAL test (E-toxate, Sigma, St. Louis, USA); this value is much lower than one that can have any endotoxic effect.

### 4.8. Flow Cytometry

A549 and DLD1 cells, “scr” and “shHsp70”, were co-cultivated with monocytic THP1 cells at a 1:1 ratio for 24 h, then THP1 cells were collected, washed, and stained on ice with antibody to arginase-1 (B&D Systems, Minneopolis, MN, USA) followed with secondary anti-sheep antibody (Abcam, Cambridge, UK) and then with third anti-rabbit antibody labeled with Alexa 647 (Invitrogen, Carlsbad, CA, USA). In order to make sure that it was Hsp70 that affects the phenotype of monocytic cells, we prepared two types of cell probes for A549 and DLD1 cancer cell lines. First, we diminished the amount of Hsp70 in the culture medium with ATP agarose (50 μL) that was added to the co-cultivation medium for the whole time of co-cultivation (probe “Hsp70(−)”). Alternatively, to increase the number of Hsp70 molecules released from the tumor cells, human recombinant Hsp70 in the concentration of 50 μg/mL was added to the A549scr and DLD1scr cell culture 18 h before introducing THP1 cells. Cancer cells were washed thoroughly from unbound chaperone [probe “Hsp70(+)”] before the addition of monocytic cells. Twenty-four hours later, THP1 cells were collected, ATP-agarose slurry were separated with the aid of Corning Cell Strainer (70 μm) from “Hsp70(−)” probe and after washing were used for western blotting analysis. The cell media from “Hsp70(+)” and Hsp70(−) probes were also collected after co-cultivation and subjected to western blotting analysis. Arginase-1-positive THP1 cells were then measured with the aid of the CytoFlex flow cytometer (Beckman Coulter, Brea, CA, USA) using a laser set at 488 (PI fluorescence) and 638 nm (Alexa647 fluorescence) and then analyzed with CytExpert 2.0 (Beckman Coulter, Brea, CA, USA) software.

Detection of apoptosis was performed using Annexin-V TM 633 (Life Technologies, Carlsbad, CA, USA) staining. A549scr and A549shHsp70 cells were co-incubated with THP1 cells at a 1:1 ratio (24 h), then THP1 cells were removed and tumor cells were treated with 20 mM etoposide for 24 h. Tumor cells were collected, washed in cold PBS, resuspended in the binding buffer provided by the manufacturer, and stained with Annexin-V-Alexa647 and propidium iodide (PI) according to the manufacturer’s recommendations. Apoptotic cells were then measured with the aid of the CytoFlex flow cytometer (Beckman Coulter, USA) using a laser set at 488 (PI fluorescence) and 638 nm (Alexa647 fluorescence) and then analyzed with CytExpert 2.0 (Beckman Coulter, USA) software.

### 4.9. Proliferation and Migration Assays

The xCELLigence system (ACEA Biosciences) provides noninvasive and label-free monitoring of cell viability, growth, and migration in real-time, based on measurement of the electrical impedance of cells adhered to an electrode on the well bottom. Increased impedance indicates that an increased number of cells adhered to the bottom at this time [57]. Two pairs of tumor cells (scr and shHsp70), namely A549 and DLD1, were incubated with THP1 cells. After 24 h of co-cultivation, THP1 cells were removed and tumor cells were placed in 16-well E-plates (4.0 × 10^4^ cells/mL; ACEA Biosciences, San Diego, CA, USA). Cell proliferation was then monitored for 48 h using the RTCA xCELLigence System. To estimate the migration capacity, we employed CIM plates according to the instructions of the manufacturer. Data analysis was performed using RTCA Analysis Software (RTCA Software Pro, xCELLigence Instruments, ACEA, San Diego, CA, USA).

### 4.10. MMPs Zymography

A549scr and A549shHsp70, DLD1scr and DLD1shHsp70, HCT-116scr, and HCT-116shHsp70 cells were coincubated with THP1 during three co-cultivation steps as described in Section 4.4. After each step, THP1 cells were removed, tumor cells were washed, culture medium was replaced with serum-free medium, and the cells were cultured for 24 h in standard conditions. Culture medium was collected after each step, centrifuged at 300× *g* to remove floating cells followed by centrifugation at 10,000× *g* for 10 min to remove cell debris. Then, 50 μL of each supernatant was mixed with 3× zymography sample buffer (60% glycerol, 0.3% SDS, 1.875 M Tris-HCl pH 6.8, and bromophenol blue), and polyacrylamide-gelatin gel electrophoresis was performed. The gels were washed twice with 2.5% Triton X-100 for 30 min and then incubated for 12 h in reaction buffer (50 mM Tris-HCl pH 7.6, 0.15 NaCl, 10 mM CaCl2, and 0.05% Brij 35) and stained with Coomassie Blue G-250 to reveal bands that corresponded to MMP-2 and MMP-9. Quantitative analysis of MMP activity was performed with the aid of TotalLab Software (CLIQS, TotalLab Ldt, Newcastle upon Tyne, UK).

### 4.11. Statistics

The data are reported as the mean ± standard error of the mean (SEM). Differences were considered to be statistically significant when *p* < 0.05 (*) or *p* < 0.01 (**).

## Supplementary Materials

Supplementary materials can be found at https://www.mdpi.com/1422-0067/21/1/59/s1.

## Abbreviations

ATP	adenosine triphosphate
CL2	chemokine (C-C motif) ligand 2
DAPI	4′,6-diamidino-2-phenylindole
eHsp70	exogenous Hsp70
EMT	epithelial-mesenchymal transition
ELISA	enzyme linked immunosorbent assay
Hsp70	heat shock protein 70 kDa
IFN-γ	interferon-gamma
IL	Interleukin
MCP-1	monocyte chemoattractant protein 1
MMP	matrix metalloproteinase
PBS	phosphate buffered saline
shRNA	small hairpin RNA
TAM	tumor-associated macrophage
TGF-β	tumor growth factor beta
TME	tumor microenvironment
TNF-α	tumor necrosis factor
