# Supplementary material for: Extracellular Hsp70 Reduces the Pro-Tumor Capacity of Monocytes/Macrophages Co-Cultivated with Cancer Cells
Source: Int J Mol Sci. 2019 Dec 20;21(1):59. doi: 10.3390/ijms21010059 (PMC6982218; doi:10.3390/ijms21010059)
Supplement: Supplementary file 1 [file ijms-21-00059-s001.pdf]

Extracellular Hsp70 Reduces the Pro-Tumor Capacity of Monocytes/Macrophages Co-Cultivated with Cancer Cells

Elena Y.Komarova, Larisa V.Marchenko, Alexander V. Zhakhov, Alina D. Nikotina, Nikolay D. Aksenov, Roman V. Suezov, Alexander M. Ischenko, Boris A. Margulis, Irina V. Guzhova

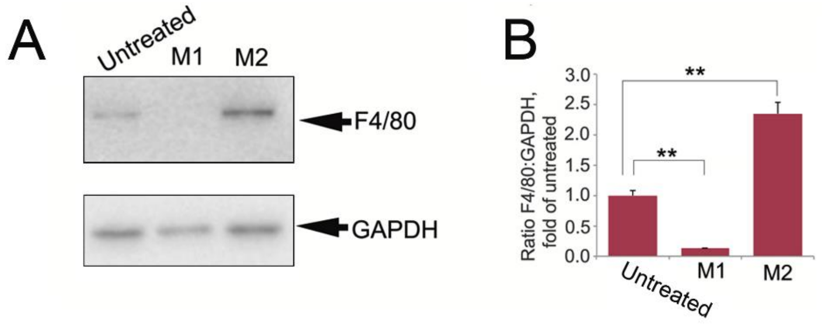

Figure 1S. *THP1 cells are able to change their phenotype under the action of certain cytokines.* A. The data of immunoblotting showed that being treated with 200 nM of phorbol myristate for 24 hours and then with 20 ng/ml of IFN- γ and 100 ng/ml LPS for 72 hours, cells approached to the M1 phenotype and significantly reduced the level of F4/80. On the contrary, after treatment with "CellXVivo Human M2 Macrophage Differentiation Kit" (R&D Systems) for 5 days the level of F4/80 in THP1 cells increased significantly suggesting that their phenotype can be regulated by tumor secretome. B. Band intensity was measured with aid of TotalLab software and ratio or F4/80 band intensity to GAPDH band intensity was calculated. **$p < 0.01$.

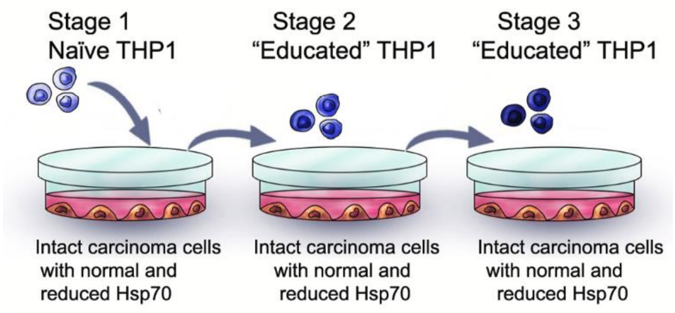

Figure 2S. The macrophage "training".

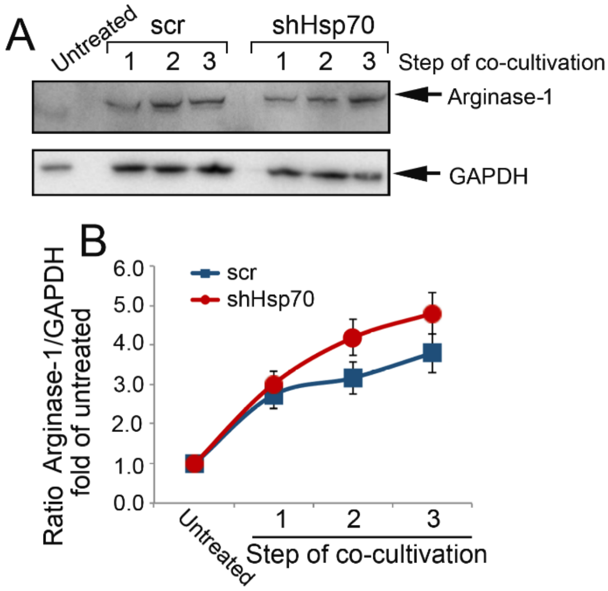

Figure 3S. Expression of pro-tumor phenotype marker, arginase-1, in THP1 monocytes upon co-cultivation with A549scr and A549shHsp70 cells increases from step to step. A. THP1 cells were incubated with A549scr or A549shHsp70 cells during 3 steps of co-cultivation. On each step the part of THP1 cell population was collected for western blotting with antibody against arginase-1. B. Band intensity was measured with aid of TotalLab software and ratio or arginase-1 band intensity to GAPDH band intensity was calculated.

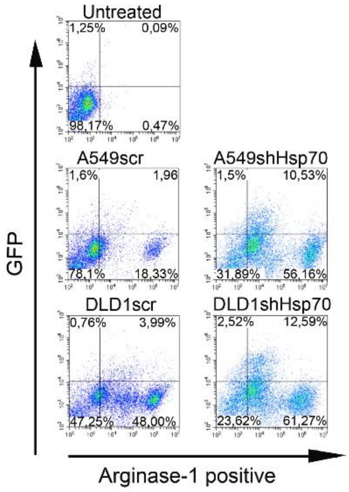

Figure 4S. Human monocytes, when incubated with tumor cells carrying a different amount of Hsp70, demonstrate pro-tumor phenotype, in a manner similar to THP1 cells. Human mononuclear fraction was separated from blood sample collected from young healthy donor with the aid Histopaque-1077 (Sigma-Aldrich,USA), and after two days in culture monocytes were transferred to A549 and DLD1 cells, “scr “and “shHsp70”, for 24 hours. Then monocytes were collected and stained with anti-arginase-1 antibody and supplied for flow cytometry assay. Since scr and shHsp70 plasmids, introduced to A549 and DLD1 cells, contained gfp gene, tumor cells detached from monolayer and contaminated monocytes fraction are reflected as GFP positive.

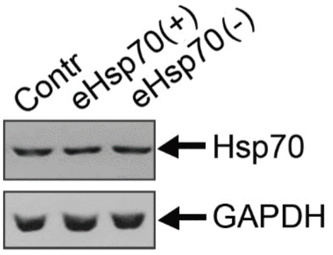

Figure 5S. Manipulations with Hsp70 in culture medium do not influence on cellular Hsp70 content. A549scr cells were incubated with pure recombinant protein Hsp70 18 hours before the introduction of THP1 cells; this manipulation caused release of endogenous Hsp70 from tumor cells («eHsp70(+)»). The cancer cells were washed three times before THP1 cells administration. In the probe «eHsp70(-)» A549scr cells were co-cultured with monocytic cells in presence of ATP-agarose. After 24 hours of co-culture A549scr cells were collected and subjected to western blotting with anti-Hsp70 antibody.
